# The cost of HIV services at health facilities in Cambodia

**DOI:** 10.1371/journal.pone.0216774

**Published:** 2019-05-29

**Authors:** Kouland Thin, Virak Prum, Benjamin Johns

**Affiliations:** 1 Health Division, Swiss Development Cooperation, Phnom Penh, Cambodia; 2 Department of Geography, Royal University of Phnom Penh, Phnom Penh, Cambodia; 3 International Development Division, Abt Associates, Inc., Bethesda, Maryland, United States; University of Ghana College of Health Sciences, GHANA

## Abstract

**Background:**

Donor funding for HIV/AIDS services is declining in Cambodia, and domestic resources need to be mobilized to sustain and expand these services. However, the cost of delivering HIV/AIDS services is not well studied in Cambodia. This study aims to assess the costs of delivering HIV/AIDS services, identify the major components of costs, and sources of funding.

**Methods:**

Four of the six highest HIV burden provinces were selected at random for this study. Within each province, four health centers and two hospitals were selected for detailed data collection. A mix of top-down and bottom-up methods were used to assess the costs for HIV testing and antiretroviral therapy (ART) from the provider perspective. We assessed the differences in the quantity and prices of inputs between health facilities of the same type to identify cost-drivers.

**Results:**

The average cost per visit for HIV testing was $8.92 at health centers and $14.03 at referral hospitals. Differences in the number of visits per staff were the primary determinant of differences in the cost per visit. First-line ART costed about $250 per patient per year, and the number of patients per staff was an important cost driver. Second-line ART costed from $500 to $716 per patient per year, on average, across the types of facilities, with the quantity and mix of second-line antiretroviral drugs being an important cost driver. Inpatient care at referral and provincial hospitals in total represented less than 2 percent of costs of outpatient ART.

**Discussion:**

Costs are similar to neighboring countries, but over 50% of the costs of ART are financed by donors. Cambodia now is scaling up social health insurance coverage; the data from this study could serve as one input when setting reimbursement rates for HIV/AIDS services to help ensure that providers are adequately reimbursed for their services.

## Introduction

One of the targets of Sustainable Development Goal 3 is to end the acquired immune deficiency syndrome (AIDS) epidemic by 2030. The number of people receiving antiretroviral therapy (ART) has risen to 17 million people from 15 million people in 2015, a target set by the United Nation General Assembly in 2011[1]. With increased ART coverage, life expectancy of people living with HIV (PLHIV) has also improved [2, 3] and the annual AIDS-related deaths has decreased by 43% [1].

Despite these achievements, huge challenges remain to end AIDS by 2030. There were around 2.1 million new infections worldwide in 2015, which increased the total number of PLHIV to about 36.7 million people. The Fast-Track approach to ending AIDS by 2030 is estimated to require about US$ 26.2 billion in 2020 [4]. The global funding for the HIV remains unpredictable [1], and donor funding for the HIV response in low- and middle- income countries (LMICs) declined after the economic crisis in 2008.

Cambodia, a country that has been internationally praised for its successes in combating the HIV epidemic [5], has, like other LMICs, also experienced funding shortages for the HIV response. Total spending for HIV in Cambodia has trended down in the past years, declining from US$ 58.1 million in 2010 [6] to US$ 40.1 million in 2016 [7]. Although domestic funding has steadily increased from US$ 4.6 million in 2009 to US$ 8.2 million in 2015, Cambodia still relies heavily on external sources of funding for HIV response [7].

HIV/AIDS programs in Cambodia have historically been financed by donors, the Ministry of Health (MoH), and out-of-pocket spending. However, in recent years Health Equity Funds (HEFs), a social insurance scheme for the poor, have begun to provide financing for some HIV/AIDS services that are not funded by the National Center for HIV/AIDS, Dermatology and Sexually Transmitted Diseases (NCHADS) [8, 9]. This provision, together with the implementation of the newly adopted national social protection policy framework 2016–2015 [10], increases the likelihood that social health insurers such as the National Social Security Fund (NSSF) will likely cover HIV/AIDS services, as has been done in other countries, including Vietnam [11, 12], Thailand [13], Mexico, and Brazil [14]. The NSSF does not clearly specify that HIV/AIDS services are included in the benefit package but the NSSF health benefit package specifies that it covers ‘treatment and care services with medical professional techniques [15].

A key factor driving provider incentives is whether, for each patient treated, the revenue brought in from various sources such as government, health insurance schemes, donors, and direct patient payments is less than, equal to, or greater than the cost of delivering those services. Revenue is largely known, but costs are not. As HEFs and perhaps NSSF take on a greater share of HIV/AIDS financing in future, cost data from health facilities can be used to shape the payment mechanisms used to purchase services and inform the rates at which HIV/AIDS services are reimbursed.

This study aims to understand the unit costs of delivering HIV/AIDS services, identify the major components of costs, and the sources of funding. The results can be used to inform HEFs or NSSF payment rates, contribute to resource estimation models, and help identify current sources of financing for HIV facility-based services.

## Methods

### Study population and sample

The study population consisted of all the health facilities (excluding national hospitals) offering HIV/AIDS services in the six provinces in Cambodia with the highest burden of HIV: Bantey Meanchey, Battambang, Kampong Cham, Phnom Penh, Siem Reap and Tbong Khum. Four of these six provinces were selected using simple random selection to be included in the study sample: Battambang, Kampong Cham, Phnom Penh, and Siem Reap. Within each province, we selected four health centers and two hospitals, including provincial and referral hospitals, using simple random sampling. In Cambodia, health centers serve as the point of primary care, typically offering general outpatient services with two or three specialized clinics (e.g., for antenatal care, normal delivery or nutrition) and 11 or fewer total staff [16]. Referral hospitals are the point of first referral, and typically have around 50 hospital beds. Provincial hospitals are the secondary point of referral, and typically can have over 100 hospital beds including specialized wards [17]. In addition, we deliberately selected one non-governmental clinic offering HIV/AIDS services in Phnom Penh for inclusion in this study. This clinic is unique in Cambodia as it offers HIV/AIDS services at two sites, but they are administratively handled together. Further, the clinic offer predominantly HIV/AIDS services targeted towards vulnerable populations, and allows for assessment of cost structures for a stand-alone HIV/AIDS service. We collected data for both sites, but report the combined costs for the two sites together.

### Services included

Costs for services, to the extent possible, were inclusive of all the inputs used to deliver services at the health facility level. The services accounted for in the costing include:

1. HIV counseling and testing: Including the costs of voluntary and confidential counseling and testing (VCCT), testing and counseling of pregnant women, and provider initiated testing and counseling (PICT).
2. Antiretroviral treatment (ART): The provision of antiretroviral therapy, including first line antiretroviral drugs (ARVs), second-line ARVs, pediatric ARVs, laboratory monitoring, and outpatient treatment of opportunistic infections(OIs). Costs were calculated for ART overall, and then broken down into costs for patients on first-line, second-line, and pediatric ART. Pediatric ART costs included the costs of early infant diagnosis of HIV. These costs did not include the costs of community-based activities.
3. Prevention of mother-to-child transmission of HIV (PMTCT): Due to difficulty in allocating costs, only costs for visits and treatment of infants exposed to HIV were included while testing of pregnant women was included in VCCT. As HIV-positive pregnant women were all enrolled in ART, costs for these services were included in ART.
4. Inpatient care for the treatment of OIs (where applicable); costs for inpatient care are not included in costs for outpatient ART and are presented separately.

### Costs included

#### Direct costs

The direct costs associated with the services at the health facility level were included in the cost analyses. These include:

1. ARVs and other drugs (primarily drugs used for OI prophylaxis or treatment) that could directly be tracked at the clinic offering HIV services within the health facility. Costs for drugs represent the amount dispensed from the health facility to ART patients, inclusive of any wastage, for outpatient care.
2. Medical supplies were included as direct costs if records of medical supply consumption were available at the relevant clinic, or staff could estimate medical supply usage. If these costs could not be obtained at the clinic level, medical supplies costs obtained at the facility level were included as an indirect cost.
3. Human resources costs for staff working directly in the clinic providing the service. These costs were based on interviews with staff estimating the amount of time they spent working in different clinics within the health facility.
4. Laboratory consumables associated with HIV/AIDS services. This included reagent and annual equivalent machine costs based on the number of tests performed for ART or VCCT patients, inclusive of CD4, viral load, alanine aminotransferase / aspartate aminotransferase, hemoglobin, creatinine, hepatitis B surface antigen, hepatitis C, and DNA polymerase chain reaction (for early infant diagnosis) tests. Note that other laboratory costs such as human resources and other overhead cost (e.g., utilities, building costs) are included in laboratory costs. These costs included the cost associated with the transport of laboratory samples to another facility, when applicable. Reagents and consumables associated with laboratory tests done off-site were included in these analyses based on estimated reagent usage per test. However, we could not include the human resources and other costs associated with conducting laboratory tests off-site due to lack of data from the referral laboratories unless the referral laboratory was located at a site included in our sample. To avoid double counting of costs, we did not include the costs of laboratory tests done off-site if the testing was referred to a type of facility that was included in the sample.
5. Ancillary services directly related to HIV/AIDS services, including partner notification, loss-to-follow-up tracking, contact tracing done by health facilities’ staff, etc.
6. HIV/AIDS-specific training, mentoring, and supervision.
7. Annual equivalent costs for equipment and furniture costs for items in the clinic where HIV/AIDS services were offered. As with human resources, in cases where HIV/AIDS services were provided in clinics with multiple functions, equipment and furniture costs were allocated to HIV/AIDS services based on the number of visits.
8. Costs for pharmaceutical products and laboratory investigations for inpatient care: In each facility where inpatient care was offered to HIV positive patients, up to 20 patient medical records for HIV were randomly sampled. In facilities with fewer than 20 admissions for a particular condition, all applicable medical records were taken. Data on the type and quantity of drugs and lab tests received were extracted from the patient medical records to estimate the costs for these categories of items. Thus, unlike for outpatient care, the costs of pharmaceutical products for inpatient care reflect the costs of prescriptions, rather than actual usage.

Cost was done from the provider perspective. Patient payments, including out-of-pocket payments at point-of-service, costs for accessing services such as payments for transportation and food expenditure were not included.

#### Indirect costs

Indirect costs included: vehicle costs, medical gases, oxygen, medical supplies, patient food, staff food, estimated cost of rent for the building, electricity, fuel and oil, cooking gas, water and water disposal, building and general maintenance, vehicle maintenance, repair and licensing, cleaning supplies and expenses, bed and linen supplies, telecommunications, office supplies and printing, insurance, meeting and visitor reception costs, uniforms, treasury tax, festival/ceremony expenses, and other indirect costs [18].

### Administrative, logistic, and ancillary clinic costs

We include costs of offices, services, and ancillary units that provide services to HIV clinics (e.g., laboratory and pharmacy units), within a health facility.

### Data collection

Six data collectors and one data collection supervisor received five days of training on the data collection tools and process starting 22 June 2017. Two data collection teams, consisting of two data collectors and one team leader, visited each facility and undertook structured data extraction. Data collection ended on 21 August 2017. The two teams each visited the same provinces at the same time, and were accompanied by a data collection supervisor who reviewed data collected each day. They interviewed staff, reviewed personnel and clinic/ward registered, reviewed drug stack cards, observed physical infrastructure, and reviewed financial records (see Annex A for an example of the data collection tool used at hospitals). Data were recorded on paper data collection forms. The paper-based data was double entered into prepared templates in Microsoft Excel, and differences between the two data entry forms were reconciled based on the paper-based data collection form. Entered data were further reviewed and corrected by research staff.

### Ethical approval

The Abt Associates Institutional Review Board approved this study on 12 May 2017, and the National Ethics Committee for Health Research (Cambodia) on 07 June 2017. The National Center for HIV/AIDS, Dermatology and STD (NCHADS) endorsed the study on 16 May 2017. Interviews of health facility staff were done only in their official capacity. The information collected from the health facilities comprised routinely collected data on utilization, supply usage, and financial records. Names, sex, age, and other direct or indirect identifying data on patients were not recorded as part of the data collection (except in cases where patients needed to be tracked between clinics and wards, in which case the identifying information did not leave the facility), nor were patients interviewed or contacted as part of this research. Summary of the research protocol for this study in English can be accessed at http://dx.doi.org/10.17504/protocols.io.2eggbbw.

### Data analysis

Data were first analyzed for each facility separately. For each facility, a Microsoft Excel workbook was set up based on the Management Accounting Systems for Hospitals template for costing health facilities [19]. The approach combines top-down (or step-down) costing with bottom-up costing of the HIV services themselves. First, indirect costs were allocated to units within the health facility, and then administrative, logistic, and ancillary clinic costs were allocated to final clinical service centers based on pre-defined allocation metrics, using a single step-down method [18]. This approach allows the ultimate total and unit costs to include both direct costs incurred in the provision of HIV/AIDS services and the relevant costs of administration, etc., allocated to the provision of HIV/AIDS services. Total costs are calculated and presented on an annual basis.

Costs are reported on average for each type of facility included in the sample, with costs for the NGO clinic reported separately from other types of facilities. Total costs are also broken down by source of financing. Unit costs reflect the total cost divided by the number of services provided. Due to the small sample sizes, ranges in total and unit costs are reported rather than with standard errors.

We assessed the differences in the quantity of inputs and the prices of inputs between health facilities of the same type (variance analysis). In order to do this, we replaced the individual input (quantity or price) of each health facility with the average amount/price of the input for that type of facility. We then recalculated the unit costs of services, and assessed the change in the average unit cost of the service. We identified all input quantities or prices that changed the average unit costs by more than 2 percent from the observed average unit costs. The items that changed the average unit costs by more than 2 percent are considered to be ‘cost drivers’–that is, inputs that potentially could be leveraged to increase the efficiency of service delivery [20]. Results are presented in 2016 US dollars.

## Results

One hospital sampled for inclusion declined to participate in the study; a replacement hospital was selected from the same province. Further, the provincial hospital in Battambang is a very large hospital, and data collection at this facility took longer than anticipated. In order to maintain data collection within the available budget, data were not collected from health centers in Battambang. Thus, the final sample included 21 health facilities: 2 provincial hospitals, 6 referral hospitals, 12 health centers, and 1 non-governmental (NGO) clinic (Table 1). None of the health centers visited offered ART or PMTCT services, while one of the referral hospitals visited did not offer VCCT services–VCCT patients were referred to a health center on-site. The NGO clinic, one referral hospital, and one provincial hospital provided pediatric ART, while four additional referral hospitals offered treatment for infants exposed to HIV.

**Table 1:** Number of facilities in sample offering selected services.

Type of facility		VCCT			ART (FL and SL)			PART		Inpatient care for OIs	Average number of FTE staff working on HIV/AIDS services (range)
	# Facilities	# Visits		# Facilities	# PYs		# Facilities	# PYs		# Facilities	
		Mean	Range		Mean	Range		Mean	Range		
Provincial Hospital	2	2,517	1,746–3,288	2	2,793	2,654–2,933	1	300	N/A	2	27 (18.6–36.0)
Referral Hospital	5	979	130–1,964	6	365	170–527	1 + 4 PMTCT only	3	N/A	5	6.4 (1.4–11.0)
Health Center	12	1,006	105–6,484	0	N/A	N/A	0	N/A	N/A	0	0.5 (0.1–1.0)
Clinic	1	3,284	N/A	1	782	N/A	1	13	N/A	0	14.3 (N/A)

Abbreviations: ART, Antiretroviral Therapy; FTE, Full Time Equivelant; OIs, Opportunistic Infections; PART, Pediartic Antiretroviaral Therapy; PYs, Person Years; PMTCT, Prevention of Mother-to-Child Transmission of HIV; VCCT, Voluntary Confidential Counseling and Testing; N/A: Not Applicable.

Results for each of the service categories (VCCT, ART, and inpatient care of OIs) are presented below. Each of these sections discusses the total cost, on average, for the provision of services at different types of health facilities, summarizes the components of the total costs, assesses the sources of financing for the total costs, presents total utilization numbers on average for the provision of services at different types of health facilities, the results for the unit costs of delivering the service, and finishes with a discussion of the variance analysis.

### Voluntary confidential counseling and testing (VCCT)

On average across the sample, the total cost of VCCT at health centers was about $4,800, ranging from $760 to $13,600 (Table 2). At referral hospitals, the total cost of VCCT was about $9,200 on average (ranging from $3,800 to $13,800); the two provincial hospitals incurred about $20,000 for VCCT. The NGO clinic had the most costs for VCCT in the sample, incurring about $45,600 in total.

**Table 2:** Total costs for VCCT services per year, average by type of facility.

Cost category	Health Center	Clinic	Referral Hospital	Provincial Hospital
Number of facilities	12	1	5	2
Building and utilities	$463	$1,711	$599	$1,114
Administrative step-down	$382	$3,329	$1,602	$2,661
Intermediate services step-down	$453	$11,428	$1,933	$3,108
Other operational indirect costs	$81	$73	$115	$559
Subtotal: Step-down / indirect costs	$1,378	$16,541	$4,250	$7,442
Salary & Benefits (clinical staff)	$2,126	$8,916	$3,278	$8,511
Equipment and furniture	$33	$2,722	$51	$296
Medical Supplies	$213	$3,342	$512	$498
Laboratory	$907	$931	$2,947	$2,947
Partner notification	$0	$0	$2	$0
Other direct activities	$0	$9,086	$0	$0
Training, mentoring, and supervision	$88	$1,829	$103	$35
Vehicles	$18	$70	$30	$164
Subtotal: Direct costs	$3,385	$29,080	$4,907	$12,451
**Total**	**$4,763**	**$45,622**	**$9,157**	**$19,893**

Abbreviations: VCCT, Voluntary Confidential Counseling and Testing.

At government-owned facilities (health centers, referral hospitals, and provincial hospitals), costs for staff were the largest category of costs, comprising over a third of costs at all levels. At the NGO clinic, ‘other direct activities’ comprised the second largest cost category at 20 percent of costs (after intermediate services step-down costs); most of these were costs incurred for a risk tracking pilot. These costs likely will change in the future as the risk tracking program matures. Costs for the rapid tests were under 20 percent of the costs, on average, across facility types.

The Royal Government of Cambodia (RGC) financed the majority of costs at government owned facilities, while the US Government funded the majority of costs at the NGO clinic (S1 Fig). External resources (The Global Fund, US Government, and other non-government sources) together provided the financing for about 11 percent to 33 percent of the costs, on average, across the types of facilities included in the sample. Health facility resources constituted about 6 percent of costs at health centers and referral hospitals, and 16 percent of costs at provincial hospitals. Generally, health facility resources are represented in medical supplies and some staff salaries–these items represent a cross-subsidy of resources from other services where user fees are charged.

Health centers and referral hospitals averaged about 1,000 visits for VCCT. However, at health centers, 81 percent of visits for VCCT were from pregnant women, while less than 10 percent of visits for VCCT at referral hospitals were from pregnant women (Table 3). PICT comprised a small (at health centers) or non-existent service at most facilities. Less than 1 percent of initial tests resulted in a confirmatory test at health centers, while over 3 percent of initial tests led to a confirmatory test at the NGO clinic and referral hospitals. At provincial hospitals, over 9 percent of initial tests were followed by a confirmatory test.

**Table 3:** Average utilization of VCCT services per year.

Service	Health Center	Clinic	Referral Hospital	Provincial Hospital
Number of facilities	12	1	5	2
Number of visits	1,006	3,284	979	2,517
of which for pregnant women	816	3	92	352
of which PICT	62	0	0	0
Average number of initial (Determine) HIV tests administered	1,007	3,284	979	2,830
Average number of confirmatory (Uni-Gold or Stapat) HIV tests administered	0.4	105	33	265

Abbreviations: HIV, Human Immunodeficiency Virus; PICT, Provider Initiated Counseling and Testing; VCCT, Voluntary Confidential Conseling and Testing.

The cost per VCCT visit costed, on average, $8.92 (range: $2.10 to $18.34) at health centers, $13.09 at the NGO clinic, $14.03 (range: $5.75 to $29.05) at referral hospitals, and $8.79 (range: $5.89 to $11.69) at provincial hospitals. Cost per initial test was similar to the cost per visit because most visits included a test.

The variance analysis showed the same two inputs were cost drivers at health centers, referral hospitals, and provincial hospitals. When the number of full time equivalent (FTE) staff per visit was changed to the average across health centers and unit costs recalculated using this average, the cost per VCCT visit changed by 19 percent (a change of $1.68 from the baseline average of $8.92), and at referral hospitals the cost per VCCT visit changed by 30 percent (a change of $4.21 from the baseline average of $14.03). At provincial hospitals, the change was 6 percent (a change of $0.49 from the baseline average of $8.79). The second cost driver was the average salary of staff, which changed unit costs by 5 percent, 2 percent, and 5 percent at health centers, referral hospitals, and provincial hospitals, respectively.

### Antiretroviral therapy (ART)

On average across the sample, the cost of ART at referral hospitals was about $104,000 (range: $31,000 to $179,000) in total (Table 4). The two provincial hospitals incurred about $740,000 (range: $583,000 to $904,000) for ART. The NGO clinic incurred about $283,000 for ART. Note that in 2016, Cambodia was transitioning to a ‘test and treat’ strategy where all patients diagnosed with HIV should start treatment immediately. However, some patients attending ART before the transition occurred, and some patients not returning until late in the year, were still classified at ‘Pre-ART’. The costs for these patients is included below, but is not reported separately because this category of costs will not be relevant in Cambodia in the future.

**Table 4:** Total costs for ART services per year, average by type of facility.

Cost category	Clinic	Referral Hospital	Provincial Hospital
Number of facilities	1	6	2
Building and utilities	$18,393	$9,989	$42,613
Administrative step-down	$17,570	$6,426	$28,659
Intermediate services step-down	$3,427	$4,888	$33,649
Other operational indirect costs	$274	$516	$5,406
Subtotal: Step-down / indirect costs	$39,664	$21,819	$110,328
Salary & benefits (clinical staff)	$38,062	$15,613	$80,149
Equipment and furniture	$4,671	$346	$709
**Pharmaceutical**			
ARV FL	$95,908	$43,081	$259,706
ARV SL	$29,116	$5,005	$99,963
Pediatric	$1,920	$346	$25,089
Other Drugs	$34,694	$7,778	$55,543
Medical Supplies	$5,386	$907	$3,198
**Laboratory**			
CD4	$6,410	$2,143	$48,464
Viral Load	$10,931	$4,077	$41,916
Other	$751	$1,303	$16,121
Sample transport	$548	$285	$144
Subtotal: Direct clinical costs	$228,397	$80,884	$631,003
Loss-to-follow-up tracking	$0	$5	$0
Other direct activities	$10,600	$49	$0
Training, mentoring, and supervision	$4,452	$742	$212
Vehicles	$264	$69	$1,916
Subtotal: Other direct costs	$15,316	$866	$2,128
**Total**	**$283,377**	**$103,570**	**$743,459**

Abbreviations: ART, Antiretroviral Therapy; ARV, Antiretroviral Drug; FL, First Line; SL, Second Line.

Cost for first-line ARVs was the largest category of costs across the facility types, ranging from 34 percent to 42 percent of total costs. At provincial hospitals, second-line ARVs were the second largest cost category (13 percent of total costs), while at the NGO clinic and referral hospitals, staff salaries and remuneration were the second largest cost category (13 percent and 15 percent of total costs, on average, respectively). All ARVs comprised 44 percent, 46 percent, and 49 percent of costs at the NGO clinic, referral hospitals, and provincial hospitals, respectively.

The Global Fund, which finances the majority of costs for CD4 and Viral Load laboratory tests and about 90 percent of the costs for ARVs and some of the drugs for treating OIs, accounted for the majority of costs across the three facility types, on average (S2 Fig). The US government provides 36 percent of the costs at the NGO clinic, with RGC contributing towards ARVs, drugs for outpatient treatment of OIs and medical supplies. NGOs provided financing at referral and provincial hospitals in the form of staff. Health facility resources contribute to less than 5 percent of the costs across facility types, on average.

At the start of the year, provincial hospitals had, on average, 2,569 patients on first line ART and 170 patients on second line ART, while referral hospitals had 350 patients on first line ART and 12 patients on second line ART. Referral hospitals had just less than 2,000 visits per year, on average, serving about 380 patients per year (Table 5). The NGO clinic had over 5,000 visits for ART and served over 1,100 patients per year, while provincial hospitals had the highest volume of ART patients, at over 16,000 visits on average in 2016, serving over 3,000 patients. In provincial hospitals, 94 percent of adult patients were on first-line ARVs, while for referral hospitals and the NGO clinic it was, 97 percent and 88 percent respectively. Patients (excluding those on pre-ART) across the types of facilities tended to have visits around every 2 months (6 visits per year). Patients at the NGO clinic and referral hospitals tended to get about 1 CD4 count test per year, and 0.6 viral load tests per year, while patients at provincial hospitals received 2.6 CD4 count tests per year, on average, and 0.8 viral load tests per year.

**Table 5:** Average utilization of ART services per year.

Service / cost category	Item	Clinic	Referral hospital	Provincial hospital
Number of facilities		1	6	2
Number of visits*		5,292	1,981	16,397
Visits by type	ARV FL	4,284	1,815	13,686
	ARV SL	586	73	1,227
	Pediatric	78	3	1,698
Number of patient-years*		1,106	382	3,079
Patient-years by type	ARV FL	690	353	2,612
	ARV SL	93	11	181
	Pediatric	13	3	300
Visits per patient-year*		4.8	5.4	5.3
Visits per patient-year by type	ARV FL	6.2	5.4	5.2
	ARV SL	6.3	5.2	7.0
	Pediatric	6.0	N/A	5.7
Laboratory	CD4	1,282	429	7,913
	Viral Load	643	240	2,343
	Other	1,484	1,171	10,796
Laboratory tests per patient per year	CD4	1.2	1.1	2.6
	Viral Load	0.6	0.6	0.8
	Other	1.3	3.1	3.5

Abbreviations: ART, Antiretroviral Therapy; ARV, Antiretroviral Drug; FL, First Line; SL, Second Line.

*Includes patients on pre-ART.

The cost per patient per year for first-line ART was $323, $263, and $214 on average for the NGO clinic, referral hospitals, and provincial hospitals, respectively (Table 6). At individual hospitals (of all types), the cost per patient per year for first-line ART ranged from $160 to $373. The cost per patient per year for second-line ART at individual facilities ranged from $417 to $976, while the cost per patient per year for pediatric ART ranged from $143 to $320 at individual facilities. The cost per visit for treating infants exposed to HIV was about $6.40 at provincial hospitals.

**Table 6:** Unit costs of ART services.

Unit	Clinic	Referral Hospital	Provincial Hospital
Number of facilities	1	6	2
Cost per visit			
First-line ART	$52.06	$50.31	$45.22
Second-line ART	$79.30	$99.16*	$108.49
Pediatric ART	$49.62	$162.73**	$28.29
ART (all)	$53.55	$50.92	$48.43
**Cost per patient per year**			
First-line ART	$323	$263	$214
Second-line ART	$501	$618*	$772
Pediatric ART	$298	$143**	$320
ART (all)	$256	$267	$240
PMTCT			
Number of facilities	0	4	1
Cost per visit for treatment of HIV exposed infants	N/A	N/A	$6.37

Abbreviations: ART, Antiretroviral Therapy; HIV, Human Immunodeficiency Viruses.

*Five referral hospitals offered second-line ART services.

**One referral hospital offered pediatric ART services.

Costs presented in this table do not include costs for inpatient treatment of opportunistic infections.

When replacing individual facility input values with the average values for provincial hospitals, none of the inputs changed the cost per person per year for first-line ARV patients by 2 percent or more. At referral hospitals, changing the number of non-CD4 and non-viral load (VL) laboratory tests changed the average cost per patient per year for first-line ARV patients by 9 percent (by $23.29 compared to a baseline average of $263.18). One referral hospital reported a high number of non-CD4 and non-VL laboratory tests compared to other facilities in the sample. Replacing the facility-specific number of patient-years per FTE with the average changed the cost for first-line ARV patients by 7 percent (by $19.18 compared to a baseline average of $263.18).

Facilities showed that patients on second-line ARVs predominantly were on an Atazanavir + Ritonavir or a Lopinavir + Ritonavir based regimen. Provincial hospitals had a higher cost per patient per year ($772) than referral hospitals ($618) for second-line ART predominantly because of different ARV mix (which accounted for 81% of the higher unit cost), with greater use of laboratory tests also contributing (accounting for 18% of the higher unit cost). At referral hospitals, the number of non-CD4 and non-VL laboratory tests is again found to be a cost-driver, again due to one facility with a high number of these types of laboratory tests. The number patients per staff, similar to patients on first-line ARVs, is also found to be a cost driver at referral hospitals, and replacing the facility-specific number of patient-years per FTE with the average changed the cost for first-line ARV patients by 5 percent.

Variation in unit costs for VCCT, first line ART, and second line ART by type of health facility and service is shown in S3 Fig. Cost varied substantially even among the same type of facility. Table 7 presents the unit cost of services by categories of input. Staff salaries and drugs were the major cost drivers.

**Table 7:** Unit cost of services by category of input.

	VCCT	VCCT	VCCT	FL ART	FL ART	SL ART	SL ART
Category of input	Health centers	Referral hospitals	Provincial hospitals	Referral hospitals	Provincial hospitals	Referral hospitals	Provincial hospitals
Salary & benefits	$4.43	$6.91	$3.70	$56.95	$25.00	$40.42	$38.74
ARV 1st line				$116.66	$100.56		
ARV 2nd line						$405.63	$548.32
Other Drugs				$16.52	$17.76	$28.13	$22.89
Medical Supplies	$0.31	$0.50	$0.19	$1.56	$1.02	$3.38	$1.33
Equipment and furniture	$0.06	$0.13	$0.12	$0.85	$0.22	$1.09	$0.32
Vehicles	$0.05	$0.06	$0.09	$0.24	$0.59	$0.21	$1.04
CD4				$6.03	$16.35	$6.56	$23.37
Viral Load Lab Test				$10.35	$13.36	$12.95	$17.72
Rapid Tests	$0.90	$0.99	$1.23				
Other Lab Tests				$5.37	$4.97	$1.06	$6.99
Sample transport				$0.93	$0.04	$0.95	$0.08
Partner notification / loss-to-follow-up tracing	$0.00	$0.00	$0.00	$0.02	$0.00	$0.01	$0.00
Training, mentoring, and supervision	$0.25	$0.06	$0.02	$1.69	$0.07	$2.35	$0.10
Indirect costs	$1.01	$1.58	$0.79	$28.14	$14.80	$34.27	$24.86
Overhead costs	$1.65	$5.87	$2.66	$33.63	$19.41	$35.22	$30.29

Abriviations: ART, Antiretroviral Therapy; ARV, Antiretroviral Drug; FL, First Line; SL, Second Line; VCCT, Voluntary Confidential Counseling and Testing.

### Inpatient care for opportunistic infections (OIs)

The average total cost of inpatient care for OIs was about $1,650 at referral hospitals and $12,300 at provincial hospitals (Table 8). In both cases, this cost was less than 2 percent of the cost outpatient ART care. Ward direct, indirect, and administrative, logistic, and ancillary clinic costs comprised 91 percent of costs, on average, at referral hospitals and 71 percent of costs, on average, at provincial hospitals.

**Table 8:** Total costs for inpatient care for opportunistic infections per year, average by type of facility.

Cost category	Referral hospital	Provincial hospital
*Number of facilities*	*5*	*2*
Pharmaceuticals	$137	$2,521
Laboratory testing	$16	$1,076
Ward direct, indirect, and step-down costs	$1,493	$8,684
**Total**	$1,647	$12,281

Referral hospitals and provincial hospitals had a similar admission rate of about 40 admissions per 1,000 ART patient-years (the NGO clinic reported that they did not refer any ART patients for inpatient care) (Table 9). Referral hospitals had substantially longer average length of stay (30.1 days per admission) than provincial hospitals (8.4 days per admission), and consequently a higher cost per admission ($266 per admission at referral hospitals versus $93 per admission at provincial hospitals) despite lower costs for drugs and laboratory procedures. Drug costs per admission averaged $17.86 per admission at referral hospitals (range: $4.53 to $40.42) and $19.00 per admission at provincial hospitals (range: $17.17 to $20.84).

**Table 9:** Average utilization and unit costs of inpatient care for opportunistic infections, average by type of facilty.

Service / Cost category	Referral hospital	Provincial hospital
Number of facilities	5	2
Average number of admittances for OIs	10	133
Number of admittances per 1,000 patient-years	37.6	43.1
Average length of stay (days)	30.1	8.4
Average cost per admission	$266	$93
Average cost per ART patient-year	$6.38	$7.97

Abbreviations: ART, Antiretroviral Therapy; OIs, Opportunistic Infections.

## Discussion

This study summarizes the cost of providing HIV/AIDS services at health facilities in Cambodia. It presents a detailed and comprehensive approach to assessing the costs of these services, including facility step-down costs and indirect costs. As such, it can serve as the basis for future resource need assessments and other financial projections assessing the costs of these services in the future. It could also be used to track how and why unit costs change over time.

### Voluntary confidential counseling and testing

The results of this study showed that the number of visits per year for VCCT was about 1,000 at health centers and referral hospitals, and the cost per visit for VCCT averaged $8.92 at health centers, and up to $14.03 at referral hospitals. The costs of providing VCCT vary from country to country, but our results suggest that costs for VCCT in Cambodia are roughly in-line with or possibly less than other countries. The costs of VCCT per client in Tanzania and Kenya were $29 and $27 in 2000, respectively [21]. The cost per complete VCCT procedure in India in 2003 was between $2.92 and $17.14 [22] whereas the cost per client was $19.26 for stand-alone VCCT and $11.68 for hospital-based VCCT Uganda in 2007 [23].

For VCCT, number of staff per visit and staff salaries were the two main cost drivers. These cost drivers reflect staff salaries and benefits as being the largest cost component of VCCT at government health facilities, and suggest that standardizing the level of effort per visit is a potential mean of increasing the efficiency of services. However, the level of effort of staff was not precisely measured in this study. At health centers especially, VCCT was delivered in other clinics (e.g., the ANC/maternity clinic) and thus variation in FTE per visit also reflects overall usage of the clinic where VCCT is delivered. While standardizing facility overhead and indirect costs does not affect the unit cost of services by more than 2 percent, it does reduce the variance in the cost per VCCT visit by over 50 percent across the three types of health facilities, again suggesting that overall facility utilization (or efficiency) is an important source of variation in the unit costs for VCCT.

### Antiretroviral therapy

The provision of first-line ART costed around $250 per patient per year, and the number of patients per staff was an important cost driver. The result is reasonably similar to the studies from Vietnam ranging from $272 [24] to $325 [25] per patient per year (although the price of some of the drugs has declined since the studies in Vietnam were conducted). The average cost per patient per year for first line ARVs was $115. Second-line ART costed from $500 to $716 per patient per year (with the ARVs costing $446 per patient per year), on average, across the types of facilities offering this service. While patients on second-line ART constituted 5.8 percent of all ART patients, they represented 14.5 percent of ART costs. The quantity and mix of second-line antiretroviral drugs was an important determinant of costs for this service. Inpatient care for OIs at referral and provincial hospitals in total represented less than 2 percent of the costs of outpatient ART.

### Financing

The results suggest that HIV/AIDS services in Cambodia are dependent on donor funding. ART is the most donor dependent, with upwards of two-thirds of all costs financed by the US Government or The Global Fund. The bulk of this (over 70% of US Government and The Global Fund support at referral and provincial hospitals) is for procurement of ARVs, and procurement for disease specific HIV drugs is largely financed by donors. Procurement of HIV commodities has been a challenge in other settings during financial transitions, and securing not just the financing, but ensuring that logistic and administrative capacity is in place may also need to be addressed in Cambodia. There is a substantial amount (e.g., 10 percent of costs at referral hospitals) of investment from the US Government in terms of staff salaries and support for ART activities. Further, all referral and provincial hospitals reported that NGO staff worked to provide ART services. Thus, securing the staff necessary to deliver ART services at hospitals will be an important issue to address when financing of ART is transitioned from external to domestic financing. For VCCT, 20 percent to 35 percent of costs are paid by donors (excluding the NGO clinic with is largely dependent on donor funding for its operation). Because VCCT is overall less expensive than ART, these funds may be easier to replace with domestic resources, but still include staff salaries (at provincial hospitals). Many ancillary support activities that are required to successfully deliver services, such as training, supervision, laboratory sample transport, loss to follow-up tracking, etc., are largely supported by donors. While not a substantial proportion of the total costs of delivering services (typically less than 5% of costs, except for at the one NGO clinic, where they constituted just over 5% of costs), ensuring their continuity during transition remains important.

### Cost drivers

There was a substantial range in the unit cost of providing services across facility types and among individual facilities. For example, the cost per patient-year for first-line ART at some referral hospitals is less than two-thirds the average cost at all referral hospitals, while at other referral hospitals it is more than 1.5 times greater than the average. Some variability in the unit costs of providing services is to be expected, given that different facilities will have different service volumes, staffing levels, etc. However, large ranges in unit costs may indicate inefficiencies in the delivery of services. Large ranges also make decisions about, for example, establishing reimbursement rates harder to accurately assess. Further work to understand the reasons for the wide ranges in unit costs and working to limit the variability in costs can help ensure that HIV/AIDS services are sustainable and help enable planning for financing of the services.

In many cases, the level of effort of staff per patient served is an important determinant of the differences in costs. Staff salaries and benefits accounted for about one quarter to nearly one half of VCCT services. At health centers, HIV services typically (but not universally) are delivered in a clinic that is not specifically for HIV (i.e., HIV services are integrated into another service, be it ANC, vaccination, or general outpatient care). In these cases, deriving efficiencies from staff effort may be difficult. However, at referral and provincial hospitals, some efficiency may be gained by rationalizing staff load (while ensuring that enough staff with the necessary skills to deliver services remain).

ARVs constitute nearly half of the costs of first-line ART, and well over half of the costs of second-line ART. As Cambodia moves to the ‘test and treat’ model of ART and more patients enroll on ART, the financing of ARVs must be accounted for. While patients on second-line ART constituted 5.8 percent of all ART patients, they represented 14.5 percent of ART costs, and second-line drugs can be up to 12 percent of the costs of delivering ART at some types of facilities. This analysis found use of second line ARVs was widely variable, on a per patient basis, across sites. Part of this variation found in this study may the result of poor record keeping, with pharmacy stock cards potentially being incomplete or inaccurate at some sites, (rather than actual differences in usage) but likely some reduction in wastage of second-line antiretroviral drugs may be possible.

Facility administration, logistics, ancillary services and indirect costs represent a substantial proportion of the overall costs for VCCT (averaging 31 percent to 46 percent of total costs across the facility types) and ART (14 percent to 21 percent of total costs across the facility types). The current financing of these costs has not been fully explored–it is likely a mix of government spending through budgetary process and block grants as well as user fee revenue. As financing of HIV/AIDS shifts, assessment of whether new payment mechanisms need to (and do) account for these costs may be necessary.

### Limitations

The results of this analysis in general, and for sources of financing in particular, may not be applicable to parts of Cambodia not included in this analysis. Donor support is variable by province and region, and more detailed understanding of what activities are done and what activities use donor support in other parts of Cambodia may be necessary. Many regions not included in this analysis have fewer persons enrolled in ART care and otherwise may experience different volumes of service, which potentially indicates that they will have different cost structures that the facilities included in this analysis. Further, data available in the registers at some sites were of questionable quality; the results of this study should be seen as indicative of the costs of services rather than a precise estimate. This study did not include costs incurred by patients to access care or the costs of NGOs operating community-based programs, and thus do not provide a holistic picture of the costs of HIV/AIDS services in Cambodia. Further work is needed to assess these aspects of costs. Further work is also needed to track how costs of HIV/AIDS health services change over time, especially as treatment protocols change and as changing sources of finance influence health service providers’ behavior. Finally, assessing in more depth how to minimize variation in costs to make financing of HIV/AIDS services more predictable would also be warranted. With the limited sample size in this study, we have not attempted to assess the potential associations between scale, maturity, geography, etc. and costs but further work in this area is warranted.

## Conclusions

The results of this study give preliminary evidence on cost of providing HIV/AIDS services at public health facilities and one NGO facility in Cambodia. Out-of-pocket payments, most notably for accessing services, and community-based costs were not included. Cambodia now is scaling up its social health insurance coverage; thus, the data from this study could be used as one input to consider when setting reimbursement rates for HIV/AIDS services assessed by insurers to help ensure that providers are adequately reimbursed for their services. Similarly, these data could serve as a basis for negotiations to contract HIV/AIDS services to private providers. In addition, these data can be used as a starting point for tracking and monitoring the cost of delivering HIV/AIDS services over time as input prices change or as efforts to improve the efficiency of service delivery are made; this is especially important because since the completion of the data collection for this study PEPFAR and Global Fund financing of HIV services has become more limited. The sample frame did not include low burden of disease provinces, and was limited in the number and type of facilities that were visited; thus, interpreting or using the data presented in this study should be done cautiously.

## Supporting information

S1 Fig Sources of financing for voluntary confidential counseling and testing. Funders include Centers for Disease Control (CDC), Non-Governmental Organizations (NGOs), the President’s Emergency Plan for AIDS Relief (PEPFAR), and the Royal Government of Cambodia (RGC).
(DOCX)

S2 Fig Sources of financing for antiretroviral therapy. Funders include Centers for Disease Control (CDC), Non-Governmental Organizations (NGOs), the President’s Emergency Plan for AIDS Relief (PEPFAR), and the Royal Government of Cambodia (RGC).
(DOCX)

S3 Fig Variation in unit costs, by type of health facility and service. (DOCX)

## References

[1] The Joint United Nations Programme on HIV and AIDS (UNAIDS). Global AIDS Update 2016. Geneva, Switzerland: UNAIDS, 2017.

[2] Slaymaker E, Todd J, Marston M, Calvert C, Michael D, Nakiyingi-Miiro J, et al How have ART treatment programmes changed the patterns of excess mortality in people living with HIV? Estimates from four countries in East and Southern Africa. Global health action. 2014;7(1):22789. 24762982 10.3402/gha.v7.22789 PMC3999950

[3] Samji H, Cescon A, Hogg RS, Modur SP, Althoff KN, Buchacz K, et al Closing the gap: increases in life expectancy among treated HIV-positive individuals in the United States and Canada. PloS one. 2013;8(12):e81355 10.1371/journal.pone.0081355 24367482 PMC3867319

[4] The Joint United Nations Programme on HIV and AIDS (UNAIDS). Fast-Track Update on Investments Needed in the AIDS Response. Geneva, Switzerland: UNAIDS, 2016.

[5] Yi S, Chhoun P, Brant S, Kita K, Suong S, Thin K, et al The Sustainable Action against HIV and AIDS in Communities (SAHACOM): Impacts on health and quality of life of people living with HIV in Cambodia. Global Journal of Medicine and Public Health 2014;3(5).

[6] Health Finance and Governance Project (HFG), National AIDS Authority (NAA). Cambodia’s Fifth National Aids Spending Assessment (NASA), 2014–15. Bethesda, MD: Health Finance & Governance Project, Abt Associates Inc, 2017.

[7] Minstry of Health (MoH). Estimating health expenditure in Cambodia: national health accounts report (2012–2016). Phnom Penh, Cambodia: Ministry of Health, 2018.

[8] Japan International Cooperation Agency (JICA), Global Link Management (GLM). Data Collection Survey on the Social Health Protection System in the Kingdom of Cambodia. Phnom Penh, Cambodia: Japan International Cooperation Agency (JICA) and Global Link Management (GLM), 2016.

[9] Allinder SM, Dattilo L. U.S. HIV Investment in Cambodia Small Program, Big Opportunity. Washington, DC: Center for Strategic and International Studies, 2017.

[10] Royal Government of Cambodia (RGC). National social protection policy framework. Phnom Penh, Cambodia: Royal Government of Cambodia, 2016.

[11] Vietnam Authority of HIV/AIDS Control HFaGP. Model to estimate health insurance liability for treatment of HIV/AIDS in Vietnam: Background, methods and results. Bethesda, MD: Health Finance & Governance Project, Abt Associates Inc, 2014.

[12] Nguyen QLT, Phan T, Tran BX, Nguyen LH, Ngo C, Phan HTT, et al Health insurance for patients with HIV/AIDS in Vietnam: coverage and barriers. BMC health services research. 2017;17(1):519 10.1186/s12913-017-2464-0 28774340 PMC5543590

[13] Ingun P, Narkpaichit C, Boongerd P. Thailand health information system improvement through universal health coverage implementation. Journal of the Thai Medical Informatics Association. 2015;2:137–47.

[14] Robin T, Humphrey E, Tomas L, TTT N. Expanding long term financing options for HIV in Vietnam. Oxford Policy Management; 2012.

[15] Ministry of Labour and Vacational Training (MLVT) and Ministry of Health (MoH). Inter-Ministerial Prakas on Provider Payment Methods for Health Care. Phnom Penh, Cambodia: MLVT and MoH, 2016.

[16] Minstry of Health (MoH). National guidelines on mininum package of activities for health center development from 2008 to 2015. Phnom Penh, Cambodia: Ministry of Health, 2008.

[17] Minstry of Health (MoH). National guidelines on complementary package of activities for referral hospital development from 2006 to 2010. Phnom Penh, Cambodia: Ministry of Health, 2006.

[18] Conteh L, Walker D. Cost and unit cost calculations using step-down accounting. Health policy and planning. 2004;19(2):127–35. 10.1093/heapol/czh015 14982891

[19] Partners for Health Reformplus. Management Accounting System for Hospitals (MASH) Manual. Bethesda, MD: The Partners for Health Reformplus Project, Abt Associates Inc, 2004.

[20] Ward WJ. Health care budgeting and financial management for non-financial managers: Greenwood Publishing Group; 1994.

[21] Sweat M, Gregorich S, Sangiwa G, Furlonge C, Balmer D, Kamenga C, et al Cost-effectiveness of voluntary HIV-1 counselling and testing in reducing sexual transmission of HIV-1 in Kenya and Tanzania. The lancet. 2000;356(9224):113–21. 10.1016/S0140-6736(00)02447-8 10963247

[22] Dandona L, Sisodia P, Ramesh Y, Kumar SP, Kumar AA, Rao MC, et al Cost and efficiency of HIV voluntary counselling and testing centres in Andhra Pradesh, India. National Medical Journal of India. 2005;18(1):26 15835489

[23] Menzies N, Abang B, Wanyenze R, Nuwaha F, Mugisha B, Coutinho A, et al The costs and effectiveness of four HIV counseling and testing strategies in Uganda. Aids. 2009;23(3):395–401. 10.1097/QAD.0b013e328321e40b 19114865

[24] Kiet PHT, Minh HV, Son NT, Binh KT. Costing HIV/AIDS Services in supported DDM Provinces in Vietnam. Bethesda, MD: Abt Associates Inc, 2013.

[25] Anh DT, Kato M, Bales S, Nhan DT, Thu NTM, Thuy CTT, et al Costing analysis of national HIV treatment and care program in Vietnam. J Acquir Immune Defic Syndr. 2014. 10.1097/QAI.0b013e3182a17d15 PMC3986422 23846564

