# Supplementary material for: The cost of HIV services at health facilities in Cambodia
Source: PLoS One. 2019 May 29;14(5):e0216774. doi: 10.1371/journal.pone.0216774 (PMC6541345; doi:10.1371/journal.pone.0216774)
Supplement: S3 Fig — (DOCX) [file pone.0216774.s003.docx]

|  | Voluntary Confidential Conseling and Testing | | | | | |  |
| --- | --- | --- | --- | --- | --- | --- | --- |
| Cost per visit (US$) |  | | | | | |  |
|  |  | | Health centers | Referral hospitals | Provincial hospitals |  |  |
|  | First line antiretroviral therapy | | | | | |  |
| Cost per patient per year | |  | | | | | |
|  |  | |  | Referral hospitals | Provincial hospitals |  |  |
|  | Second line antiretroviral therapy | | | | | |  |
| Cost per patient per year |  | | | | | |  |
|  |  | |  | Referral hospitals | Provincial hospitals |  |  |
