# Supplementary material for: The cost of HIV services at health facilities in Cambodia
Source: PLoS One. 2019 May 29;14(5):e0216774. doi: 10.1371/journal.pone.0216774 (PMC6541345; doi:10.1371/journal.pone.0216774)
Supplement: Supplementary file 3 [file pone.0216774.s002.docx]

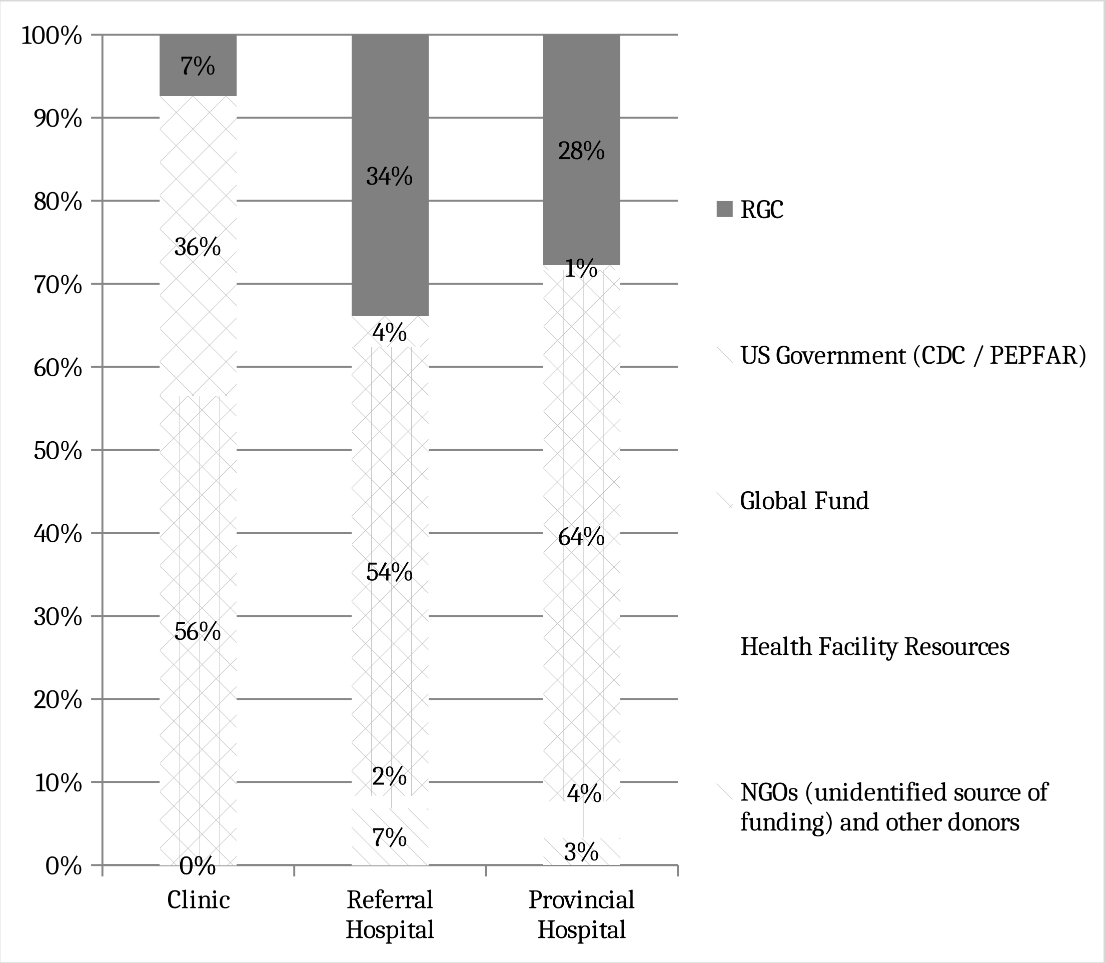
100%
90%
80%
70%
60%
50%
40%
30%
20%
10%
0%
7%
36%
56%
0%
34%
4%
54%
2%
7%
28%
1%
64%
4%
3%
Clinic
Referral Hospital
Provincial Hospital
RGC
US Government (CDC / PEPFAR)
Global Fund
Health Facility Resources
NGOs (unidentified source of funding) and other donors
